# Why an Orthopædic Department Is Necessary

**Published:** 1913-11-01

**Authors:** 


### THE ORTHOPÆDIC DEPARTMENT.

### I.—Why an Orthopaedic Department is Necessary.

Although orthopædics is not a modern development of surgery, it is only during the last half of the nineteenth century that it has attained to the dignity of a recognised speciality. Previous to the pioneering efforts of the French surgeons, who devoted their attention and genius to perfecting certain methods of treatment employed in cases of deformity, orthopædics had no distinct standing. The general surgeon, who admitted the claim of the throat and nose specialist, who took it for granted that it was right and proper that a general hospital should make provision for the services of an ophthalmologist, and who, reluctantly enough it is true, agreed to divide his chirurgical responsibility in certain cases with a neurologist, an aurist, or a gynæcologist, refused point blank to tender to the orthopædist the same degree of recognition. The result of this conservative policy, which is still followed in some of our older hospitals, was that the general surgeon found himself compelled to deal with diseases that had become, as it were, part of the domain of a speciality still struggling for its rights. A certain amount of unavoidable antagonism was developed on both sides, which, in turn, led to a certain amount of unavoidable overlapping in treatment. It is not our intention to point out in detail how such overlapping arose, or to show in what particulars conservative surgery and struggling orthopædy were to blame for the retardation of the art of treating deformities that resulted from such antagonism. Happily to-day most of us, surgeons as well as physicians, are agreed that orthopædy is a speciality that demands special aptitude on the part of the surgeon, special knowledge and skill obtained by frequent practice, special facilities, and special technique.

We no longer refuse to acknowledge the claims of the orthopædic surgeon, for he has proved his case up to the hilt. Unfortunately, surgery has lost something in the dispute that preceded this whole-hearted recognition of claims that ought to have been conceded long ago. The public has come to believe, with qualifications, in the bone-setter, who is virtually an empiric orthopædist—often, let us hasten to add, a highly skilled, knowledgeable empiric—who, through constant practice, has attained a perfection in technique which the orthopædist would be the last to deny—but it still fights somewhat shy of the orthopædist. The reason for this shyness is obvious. So long as general surgeons continue to dabble in a speciality which they have not studied and in which they have not perfected themselves, so long must they expect their results to be inferior, so far as bloodless surgery is concerned, to those obtained by the bone-setter. And the public judges by results. It overlooks entirely the general knowledge that the surgeon possesses and that safeguards him from the slips that the bone-setter not infrequently makes. It is well to realise this attitude on the part of the public. Hospital workers and committee men especially should pay some consideration to it and should see that the institution with which they are associated is sufficiently up to date and progressive to recognise the value of the orthopædic department as an integral part of the combination of perfect units that make up the modern hospital.

To some this recognition may yet appear to be superfluous. It may be argued that orthopædy is not a fixed speciality as is ophthalmology, or even gynæcology. But it is well to bear in mind that all specialities in medicine are bound to overlap, and that it is an axiom that a specialist in any department must have a general knowledge of surgery or medicine and be able to correlate his special interests with those of his colleagues. Orthopædy, properly speaking, is no longer the art of correcting deformities of the lower extremity; it has broadened into the art of correcting all deformities, and the only difficulty now is to define what, strictly speaking, constitutes deformity. There are some deformities, acquired and congenital, which by common consent have been relegated to the general surgeons' department. These are, to quote a few salient examples, herniæ, tumours, cleft palate, and hare-lip. But it is argued by some orthopædists that all deformities fall under their category and should be dealt with in the special department. This claim is too broad to be generally admitted. For the present at least orthopædy, as a speciality, must concern itself more particularly with such deformities as affect the patient's gait and the movements of trunk or limbs. It is easy to see that, even with this limitation, the least that the orthopædist will admit and the most that the general surgeon will concede, there must be a certain amount of overlapping which cannot be avoided. Similar overlapping occurs in other special departments, and by itself is no reason against the establishment of a special department. For the justification of a special department is this—that in it certain patients can be treated better and with greater thoroughness and, consequently, the hope of greater ultimate success, than in the general wards.

To come from the abstract to the concrete, let us take so familiar a hospital case as a patient afflicted with lateral curvature. Such a patient cannot be adequately treated in the general outpatient department of a hospital, nor in a general ward. His condition is a chronic one that demands for its relief and cure special treatment, special apparatus, and special attention which, in the majority of cases, the general surgeon cannot give. Such a case, therefore, is admirably suited for a special department, and it is from such cases that the orthopædic department will draw its patients. The modern hospital, in order to be able to deal with such patients, should have a special unit in which adequate provision is made for the installation of such necessary apparatus as is required for the treatment of static deformities by up-to-date methods. In the following articles we shall try briefly to outline what these requirements are. At present it is sufficient to draw attention to the fact that special requirements are necessary and that the establishment of a special department is not only justifiable in institutional interests, but is absolutely essential if the hospital wishes to be considered up to date and efficient.

This position has been considered by some of our general hospitals and arrangements have already been made to realise in some ways the ideal. In London the London Hospital started with its excellent orthopædic department, which is to-day one of the best in the kingdom, although it cannot be said to be absolutely perfect. St. Bartholomew's followed suit with a department which may yet become the best in the Metropolis, and lately Guy's has also seen the advisability of having its own orthopædic department and has started to establish one. The combination of the various City orthopædic hospitals into one establishment, the Royal National Orthopædic Hospital, in Great Portland Street, has enabled that institution to become one of the best and most efficient orthopædic hospitals in the British Isles. Charing Cross Hospital and the Children's Hospital in Great Ormond Street also possess good orthopædic departments, though neither can lay claim to the perfection that characterises similar departments in Continental or American institutions. In the provinces similar advances have been made, especially in Liverpool, where the pioneering work of Robert Jones has succeeded in establishing what may justifiably be called the British School of Orthopædy.

In the circumstances it is not unlikely that in the near future every hospital, including children's hospitals, will follow the example of the larger Metropolitan institutions and establish such useful and desirable units as the orthopædic department. Modern advance in medicine and surgery has made such progress imperative for all hospitals that wish to be considered efficient, and no one can afford to lag behind the other in the striving to give to its patients the best treatment and the most up-to-date methods of cure that modern science holds out.

